# Bioinformatics and Network Pharmacology-Based Approaches to Explore the Potential Mechanism of the Antidepressant Effect of Cyperi Rhizoma through Soothing the Liver

**DOI:** 10.1155/2021/8614963

**Published:** 2021-12-28

**Authors:** Yuhe Lei, Mingquan Du, Ge Zhang, Lei Chen, Yanli Fu, Yinqin Zhong, Enxin Zhang

**Affiliations:** ^1^Department of Pharmacy, Shenzhen Hospital of Guangzhou University of Chinese Medicine, Shenzhen 518032, Guangdong, China; ^2^Department of Pharmacy, Futian Center for Chronic Disease Control, Shenzhen 518000, Guangdong, China; ^3^Chinese Medicine Development Research Center, Guangdong Provincial Hospital of Chinese Medicine, Guangzhou 510120, Guangdong, China; ^4^Department of Oncology, Shenzhen Hospital of Guangzhou University of Chinese Medicine, Shenzhen 518032, Guangdong, China; ^5^Shenzhen Hospital of Guangzhou University of Chinese Medicine, Shenzhen 518032, Guangdong, China

## Abstract

Major depressive disorder (MDD) has become the second most common disease worldwide, making it a threat to human health. Cyperi Rhizoma (CR) is a traditional herbal medicine with antidepressant properties. Traditional Chinese medicine theory states that CR relieves MDD by dispersing stagnated liver qi to soothe the liver, but the material basis and underlying mechanism have not been elucidated. In this study, we identified the active compounds and potential anti-MDD targets of CR by network pharmacology-based approaches. Through Gene Ontology (GO) and Kyoto Encyclopedia of Genes and Genomes (KEGG) enrichment analysis, we hypothesized that the anti-MDD effect of CR may be mediated by an altered response of the liver to lipopolysaccharide (LPS) and glucose metabolism. Through bioinformatics analysis, comparing normal and MDD liver tissue in rats with spontaneous diabetes, we identified differentially expressed genes (DEGs) and selected PAI-1 (SERPINE1) as a target of CR in combating MDD. Molecular docking and molecular dynamics analysis also verified the binding of the active compound quercetin to PAI-1. It can be concluded that quercetin is the active compound of CR that acts against MDD by targeting PAI-1 to enhance the liver response to LPS and glucose metabolism. This study not only reveals the material basis and underlying mechanism of CR against MDD through soothing the liver but also provides evidence for PAI-1 as a potential target and quercetin as a potential agent for MDD treatment.

## 1. Introduction

Major depressive disorder (MDD), a complicated mental disease, affects approximately 340 million people, and 1 million people die each year due to suicide [1]. MDD is characterized by multiple emotional and physical disorders, such as depressive mood, excessive guilt, insomnia, poor concentration, and suicidal ideation [2]. Various factors contribute to the initiation of MDD, including neuronal apoptosis, decreased neurotransmitter secretion, psychosocial factors, and abnormal immune and endocrine systems [3]. Recent research demonstrated that diabetes and inflammation are both key risk factors for MDD [4, 5]. The levels of numerous proinflammatory cytokines, such as interleukin-6 (IL-6), interleukin-1B (IL-1B), and tumour necrosis factor-*α* (TNF-*α*), are elevated in MDD patients [6, 7]. It has been reported that lipopolysaccharide (LPS) induces systemic inflammation by elevating proinflammatory cytokines in both the periphery and brain, thus resulting in depression-like behaviour [8]. Additionally, epidemiological studies have shown that depression is twice as frequent in people with diabetes compared with those without diabetes [9]. Although the mechanism of diabetes-induced MDD is not completely understood, metabolic disorders, such as persistent hyperglycaemia in diabetes, can change the function, neurochemistry, or structure of the brain to mediate MDD [10]. LPS and glucose metabolism are closely related to the liver, a central organ for systemic metabolism [11]. The liver is responsible for the clearance of LPS in the blood [12]. As an organ of action for insulin, the liver regulates blood glucose by producing glucose during fasting and storing glucose postprandially [13]. In type 1 and type 2 diabetes mellitus, hepatic processes are dysregulated and contribute to hyperglycaemia [14]. In brief, the liver may play a vital role in LPS and glucose metabolism to mediate MDD.

Cyperi Rhizoma (CR), a traditional herbal medicine, has been clinically used for menstrual or emotional disturbances in women and stomach disorders in Asia for centuries [15]. Modern pharmacological research has revealed the anti-inflammatory, antiapoptotic, and antibacterial properties of CR [16]. Recent studies have demonstrated that CR possesses therapeutic effects on nervous system diseases, such as 6-hydroxydopamine-induced neuronal damage [17]. In addition, CR extract exerts an antidepressant effect on mice characterized by shorter immobility time in the swimming test and the tail suspension test [18, 19]. Traditional Chinese medicine (TCM) theory states that CR relieves depression and anxiety by dispersing stagnated liver qi to soothe the liver. However, the material basis and antidepressant mechanism of CR have not been well established.

Plasminogen activator inhibitor-1 (PAI-1, SERPINE1) is a prothrombotic plasma protein secreted by endothelial tissue [20]. As a stress-related protein, PAI-1 has been implicated in numerous disease states, including MDD. Elevated plasma levels of PAI-1 have been observed in depressed patients and C57Bl/6J mice [21]. Another meta-analysis also demonstrated that the PAI-1 serum level was 0.27 SDs higher in MDD patients than it was in healthy controls [22]. Abnormal PAI-1 causes MDD through different mechanisms. First, PAI-1 may contribute to the cleavage of pro-brain-derived neurotrophic factor (BDNF) into its mature form, and BDNF is strongly implicated in depression [23]. In addition, PAI-1 is widely regarded as an inflammatory marker involved in the pathogenesis of depression [24]. PAI-1 participates in LPS-induced inflammation [25]. Inhibition of PAI-1 by histone deacetylase 2 (HDAC2) attenuates LPS-induced inflammation [26]. Furthermore, the PAI-1 levels were significantly higher among patients with metabolic syndrome, and 53.2% of these patients experienced depression [24]. For example, an elevated level of PAI-1 was detected in diabetes- and insulin-resistant states, which is associated with an imbalance in glucose and lipid homeostasis [27]. Given that the liver is a major site for PAI-1 synthesis and responds to a variety of hormonal changes and other pathological events [28], it can be deduced that liver disorders and MDD are closely associated.

In our present study, we selected active compounds and identified PAI-1 as a target of CR to combat MDD by regulating the liver response to LPS and glucose metabolism, which may shed light on the modern pharmacological connotation of the antidepressant effect of CR through soothing the liver.

## 2. Materials and Methods

### 2.1. Compounds and Targets Screening of CR

Chemical compounds and their related targets were collected from the Traditional Chinese Medicine Systems Pharmacology (TCMSP) database (https://old.tcmsp-e.com/tcmsp), a unique pharmacology platform for Chinese herbal medicines. To further obtain the active compounds according to the ADME (adsorption, distribution, metabolism, excretion) parameters, we chose compounds meeting the requirements of both oral bioavailability (OB) ≥ 30% and drug-likeness (DL) ≥ 0.18. OB is a vital indicator of the orally administered dosage of unchanged drug entering the human circulatory system [29]. DL was applied to filter out compounds with undesirable properties [30]. To obtain the gene symbol of each target, we used UniProt (https://www.uniprot.org) to convert protein names to gene symbols.

### 2.2. Screening of Potential Targets of CR in the Context of MDD

GeneCards (http://www.genecards.org/) was used to search for the therapeutic target genes of MDD. Key terms related to “major depressive disorder” were retrieved, and the requirement of relevance score ≥10 was set. We selected genes at the intersection of CR and MDD (C&M) as potential target genes through which CR exerts effects on MDD, and the corresponding compounds of CR were regarded as candidate components with antidepressant properties.

### 2.3. Network Construction

The drug-compound-target-disease network was established using the network visualization software Cytoscape (v3.8.2; Agilent Technologies Company, USA). Each node in the network represents a medicine, disease, target, or compound. If an interaction occurred between nodes, they were connected by a line. Topology analysis was achieved in CentiScaPe.

### 2.4. Protein-Protein Interaction (PPI) Network Construction

The PPI network of C&M targets was built using STRING version 11.0 (https://www.string-db.org/). The targets of C&M were entered into the STRING database. The species was limited to *Homo sapiens*, and an interaction score greater than 0.4 was required. The bitmap image and network information were downloaded from this website. After enrichment of all the nodes, we obtained the top 30 targets that were considered to play central roles in the PPI network.

### 2.5. Gene Ontology (GO) and Kyoto Encyclopedia of Genes and Genomes (KEGG) Pathway Enrichment

We conducted GO and KEGG enrichment analysis based on Bioconductor software (http://bioconductor.org/). GO enrichment included the biological process (BP), molecular function (MF), and cellular component (CC) categories. We used clusterProfiler (version 4.1), an *R* package, to perform the GO and KEGG enrichment analysis of gene clusters. The results were considered significant when the *P* value < 0.05. The top 5 GO and KEGG enriched results are displayed in the bar plot.

### 2.6. Identification and Analysis of Differentially Expressed Genes (DEGs)

The microarray data set GSE94988, which includes 3 control samples, 3 mild depression samples, and 3 major depression samples, was downloaded from the Gene Expression Omnibus (GEO) database (http://www.ncbi.nlm.nih.gov/geo/) using the GEOquery package (version 2.54.1) [31]. In the original research, the analysis of GSE94988 aims to explore the mechanism of pharmacokinetic perturbation in a CUMS-induced depression animal model with spontaneous diabetic GK rats [32]. The expression profile in GK rat livers was screened using Affymetrix Rat 230 2.0 Array. The original files from the GEO database were normalized by the *R* software (version 4.0.2) affy package. DEGs between 3 control samples and 3 major depression samples were screened using the limma package (version 3.42.2), which can fit a linear model for the expression of each gene. The screening criteria were *P* < 0.05, and |log2-fold change (FC)| > 1 between the two groups. To better visualize these DEGs, volcano plots were made using the ggplot2 package (version 3.3.3), and heatmaps were generated using the ComplexHeatmap package (version 2.2.0) [33]. Venn diagrams were used to display the intersection between DEGs and C&M targets.

### 2.7. Molecular Docking between Targets and Compounds

The crystal structure of the PAI-1 protein (PDB id: 7AQF) was downloaded from RCSB PDB (https://www.pdbus.org/). GUI-based “AutoDock Tools” were used to prepare and execute the docking studies. Kollman atom charges, solvation parameters, and polar hydrogens were added to the protein, and this information was used for docking studies. As the ligands used are not peptides, Gasteiger charges were assigned only to the protein, and nonpolar hydrogens were merged. Based on the literature and predicted active regions, a grid box was assigned around the active sites using the AutoGrid application. The 3D structures of quercetin were retrieved from the PubChem database (https://pubchem.ncbi.nlm.nih.gov). Then, we minimized the energy of the downloaded compound through Chem3D and converted it into mol2 format. The small molecular compound was imported into AutoDock Tools software, added with atomic charge, and assigned an atomic type. All flexible keys are rotatable by default. Finally, the best conformation was retained in pdbqt format for utilization in further docking studies. Docking calculations were performed using AutoDock 4.2 to compute the free energy of binding in the protein model. Essential hydrogen atoms, Kollman united atom type charges, and solvation parameters were added with the aid of AutoDock tools. Affinity (grid) maps of 60 × 60 × 60 Å grid points and 0.375 Å spacing were generated using the AutoGrid program. The AutoDock parameter set and distance-dependent dielectric functions were used in the calculation of the van der Waals and electrostatic terms, respectively. Docking simulations were performed using the Lamarckian genetic algorithm (LGA) and the Solis and Wets local search method. The initial position, orientation, and torsions of the ligand molecules were set randomly, and all rotatable torsions were released during docking. Each docking experiment was derived from 10 different runs that were set to terminate after a maximum of 250,000 energy evaluations. The population size was set to 150. During the search, a translational step of 0.2 Å and quaternion and torsion steps of 5 were applied.

### 2.8. Molecular Dynamics

The molecular dynamics (MD) simulation of docked complexes was performed using Desmond version 2020. Here, an OPLS3e force field was used to initiate the MD simulation, and the system was solvated using the TIP3 water model. The neutralization of the system was performed by adding counter ions. Energy minimization of the entire system was performed using OPLS3e because it is an all-atom type force field. The geometry of water molecules, the bond lengths, and the bond angles of heavy atoms were restrained using the SHAKE algorithm. Simulation of the continuous system was executed by applying periodic boundary conditions, and long-range electrostatics were maintained by the particle mesh Ewald method. The equilibration of the system was performed using an NPT ensemble with temperature at 300 K and pressure at 1.0 bar. The coupling of temperature-pressure parameters was performed using the Berendsen coupling algorithm. After preparation of the system, the production run was performed for 100 ns with a time step of 1.2 fs, and trajectory recording was performed every 100 ps summing up to the recording of 1,000 frames. The root mean square deviation (RMSD) was calculated for the backbone atoms and was analysed graphically to understand the nature of protein-ligand interactions. The root mean square fluctuation (RMSF) for every residue was calculated to understand the major conformational changes in the residues in comparison between the initial state and dynamic state.

### 2.9. Statistical Analysis

The results are presented as the mean ± standard deviation (SD). Statistical analysis was performed using GraphPad Prism 7.0 (GraphPad Software Inc.) and ImageJ software (National Institutes of Health, USA). The differences between groups were evaluated by one-way analysis of variance (ANOVA) followed by Tukey's post hoc test. *R* software was also used to perform statistical analyses. *P* < 0.05 was considered statistically significant.

## 3. Results

### 3.1. Active Compounds and Targets of CR

Given that herbal medicine is characterized by the use of multicomponent and multitarget therapeutic drugs, it is necessary to select active compounds with satisfactory pharmacokinetic properties for further research. After retrieval in TCMSP, a total of 104 compounds from CR were obtained. Based on the ADME model, we selected 18 active compounds that meet the requirements of both oral bioavailability (OB) ≥ 30% and drug-likeness (DL) ≥ 0.18 (Table 1). Information on compound-related targets was also obtained from TCMSP. Ultimately, the 18 active compounds and 225 corresponding targets constitute 496 compound-target connections. The official gene symbols of targets were obtained from the UniProt database for further investigation.

### 3.2. Potential Anti-MDD Targets of CR

**Figure 1:**
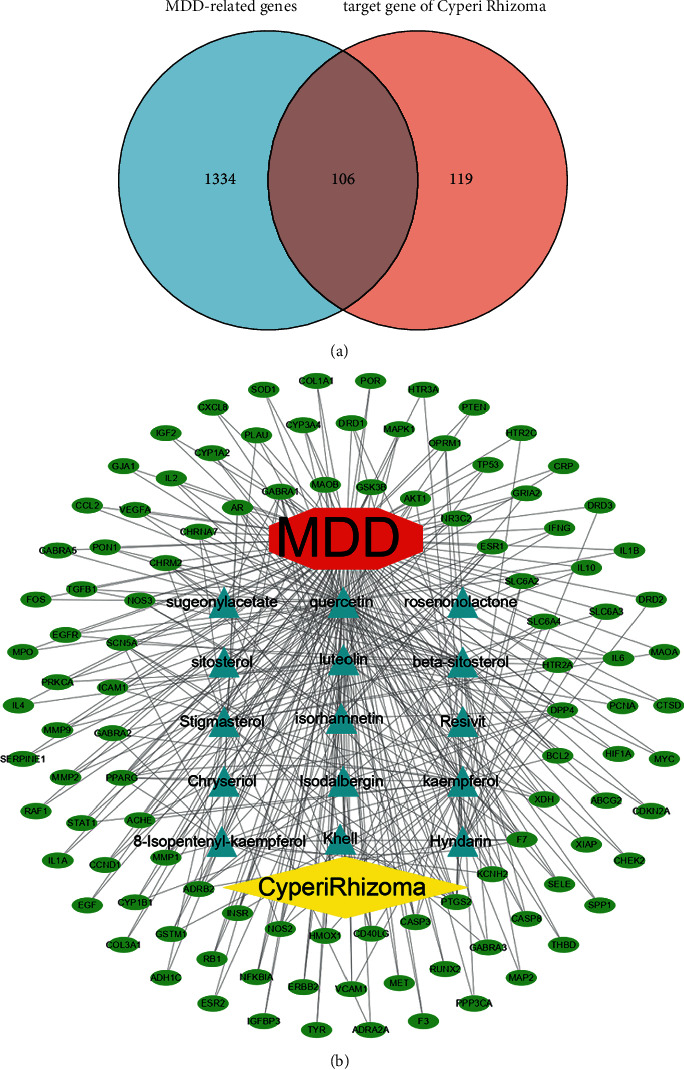
Screening of potential effective ingredients and anti-MDD targets of CR. (a) Venn diagram of MDD-related targets and potential targets of CR. (b) The drug-compounds-targets-disease network. The red octagon represents disease. The yellow rhombus represents the drug. The blue triangles represent active compounds. The green ovals represent target genes.

To further predict the potential targets of CR against MDD, a drug-compound-target-disease network was established to analyse the mechanism. The GeneCards database was used to retrieve MDD-related therapeutic targets. As a result, 10,026 MDD-related targets were obtained, and 1440 targets that met the requirement of a relevance score ≥10 were selected. A Venn diagram (Figure 1(a)) showed the overlap of a total of 106 significant targets between CR and MDD (C&M), indicating that 106 potential targets were involved in the anti-MDD effect of CR. The 15 compounds corresponding to 106 targets were regarded as candidate effective components of CR. Next, we applied Cytoscape software to construct a drug-compound-target-disease network. As shown in Figure 1(b), 15 drug-compound, 494 compound-target, and 106 target-disease connections were created in a network. The network map displays the synergistic effect of multiple ingredients of CR as they converge on multiple targets. To preliminarily screen key targets in the C&M network, 106 potential targets were collected for further analysis.

### 3.3. PPI Analysis of C&M Targets

**Figure 2:**
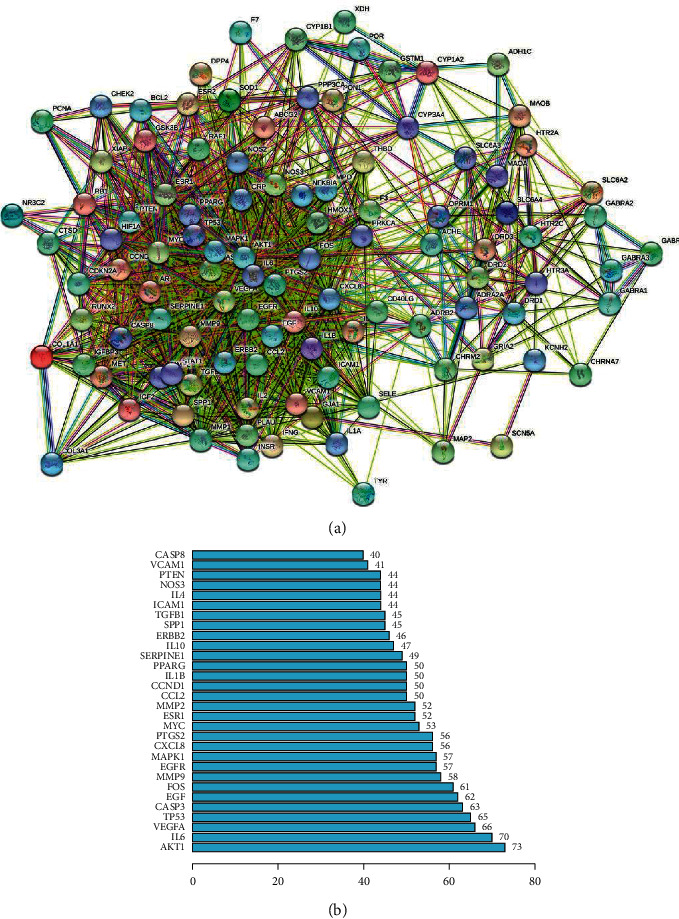
Preliminary screening of key C&M targets. (a) PPI network of C&M targets. Each node represents the C&M targets. Each line represents the interaction between two targets. (b) Bar plot of top 30 enriched targets in the PPI network.

The STRING database was used to establish the PPI network of C&M targets. A total of 106 target genes of C&M were submitted to the STRING website, and 1543 connections that represent the interaction between two targets were generated (Figure 2(a)). To screen core targets thoroughly, the frequency of each node and the combined score between two nodes were calculated. A bar plot (Figure 2(b)) shows the top 30 enriched targets, which may represent the most likely targets of CR against MDD. The interaction nodes of higher degree include AKT1, IL6, VEGFA, and TP53, indicating that these nodes are associated with more proteins and may play pivotal roles in the antidepressant effect of CR.

### 3.4. GO and KEGG Enrichment Analysis

**Figure 3:**
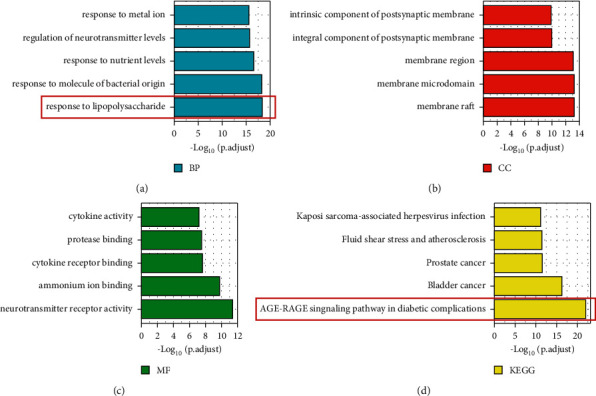
The 5 most representative items identified by GO and KEGG enrichment. (a) Blue columns represent the BP category of GO. (b) Red columns represent the CC category of GO. (c) Green columns represent the MF category of GO. (d) Yellow columns represent the KEGG pathway enrichment. The columns that are significant and selected for further research were framed.

To clarify the anti-MDD mechanism of major active compounds of CR, GO and KEGG enrichments were conducted based on 106 C&M targets.  Table 2 provides details of GO and KEGG enrichment analysis.  Figure 3(a)–3(d) display the top 5 enriched GO (BP, MF, and CC terms) and KEGG pathways. The results demonstrate that some items are closely related to the nervous system, such as “regulation of neurotransmitter levels,” “intrinsic component of postsynaptic membrane,” “integral component of postsynaptic membrane,” “ammonium ion binding,” and “neurotransmitter receptor activity,” indicating that CR displays antidepressant effects through multiple biological processes and signalling pathways. Interestingly, we noticed that “response to lipopolysaccharide” ranks top in the BP category, and the “AGE-RAGE signalling pathway in diabetic complications” ranks top in KEGG pathway enrichment. It has been reported that LPS and diabetes are both risk factors in MDD due to metabolic dysfunction [34, 35]. TCM states that CR disperses stagnated liver qi to relieve depression [36]. Additionally, the liver was verified to play significant roles in LPS and glucose metabolism [37, 38]. Based on these facts, we hypothesized that CR displays antidepressant effects by regulating liver functions to combat LPS and glucose metabolic disorders. As a consequence, we collected enriched “response to lipopolysaccharide” and “AGE-RAGE signalling pathway in diabetic complications” gene sets for further investigation.

### 3.5. Identification of DEGs

**Figure 4:**
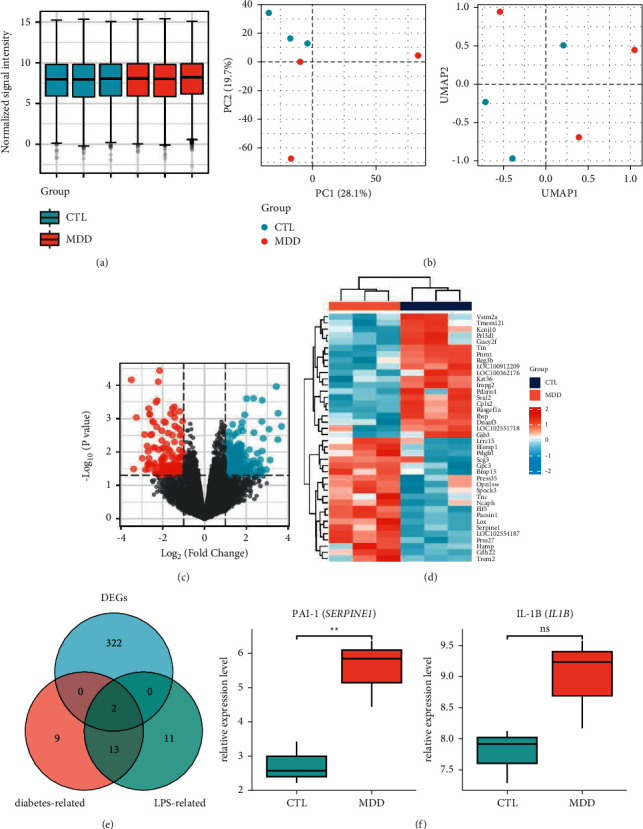
Identification of potential targets of CR in combating MDD through modulating the liver response to LPS and glucose metabolism. (a) Normalization of 3 control samples and 3 MDD samples. (b) Principal component analysis (PCA) and uniform manifold approximation and projection (UMAP) before DEG analysis. PCA and UMAP were applied to 3 MDD liver tissues (red) and 3 CTL liver tissues (blue) characterized by the gene expression of all probes on Affymetrix Rat 230 2.0 Array. (c) Volcano diagrams of MDD-related DEGs. Red dots represent upregulated DEGs, and green dots represent downregulated DEGs. (d) Heatmaps of MDD-related DEGs. The colour shift from blue to red indicates a trend from low to high expression, respectively. (e) Venn diagram of DEGs, LPS-related genes, and diabetes-related genes. (f) Relative expression levels of PAI-1 and IL-1B in the MDD group compared with the control group. *∗∗P* < 0.01 vs. control.

To confirm our hypothesis, we identified the DEGs of liver tissue between the normal group and the MDD group. We obtained the microarray data set GSE94988, which includes 3 control samples, 3 mild depression samples, and 3 major depression samples. All the 9 samples have spontaneous diabetes. Three control samples and 3 MDD samples were selected to analyse and identify the DEGs. Before analysis, the original data were preprocessed and normalized based on the *R* software affy package.  Figure 4(a) indicates good normalization of the 6 samples. Quality control of the samples was assessed by principal component analysis (PCA) and manifold approximation and projection (UMAP), which can evaluate the intragroup data repeatability and display the relationships between the groups of samples that were compared. PCA results (Figure 4(b)) demonstrated that the first two principal components, principal component 1 (PC1) and principal component 2 (PC2), accounted for 28.1% and 19.7% of the explained variation, respectively. In PCA and UMAP plots, samples were scattered between the CTL and MDD groups, indicating significant differences between the two groups. Thus, subsequent analysis may produce more meaningful results. Compared with control samples, a total of 324 DEGs were identified in MDD samples using the criteria of *P* < 0.05 and |log2FC| > 1. These 324 DEGs included 142 downregulated genes and 182 upregulated genes. Volcano diagrams of DEGs are presented in Figure 4(c). The heatmaps in Figure 4(d) display the top 20 downregulated and upregulated genes. These DEGs may be potential targets of the liver to mediate MDD in diabetic rats.

### 3.6. Potential Targets of CR on LPS and Glucose Metabolism to Combat MDD

To determine the most likely targets of CR in combating MDD through modulating the liver response to LPS and glucose metabolism, we focused on the overlaps between 26 “response to lipopolysaccharide” genes, 24 “AGE-RAGE signalling pathway in diabetic complications” genes, and 324 DEGs. A Venn diagram (Figure 4(e)) displays the overlap of 2 significant targets, namely, SERPINE1 and IL1B, indicating that PAI-1 (SERPINE1) and IL-1B (IL1B) may be potential targets of CR regarding LPS and glucose metabolism to combat MDD. SERPINE1 and IL1B were also listed in the top 30 enriched targets in the PPI network (Figure 2(b)), indicating their central regulatory roles in the antidepressant effect of CR. PAI-1 and IL-1B expression levels were evaluated by GSE94988 data analysis. As shown in Figure 4(f), PAI-1 was significantly upregulated in the MDD group (*P* < 0.01), whereas IL-1B was not significantly different (*P* > 0.05). Hence, we selected PAI-1 as the most likely target of CR to combat MDD, and the corresponding active compound was quercetin.

### 3.7. Molecular Docking between the Target and Compound

**Figure 5:**
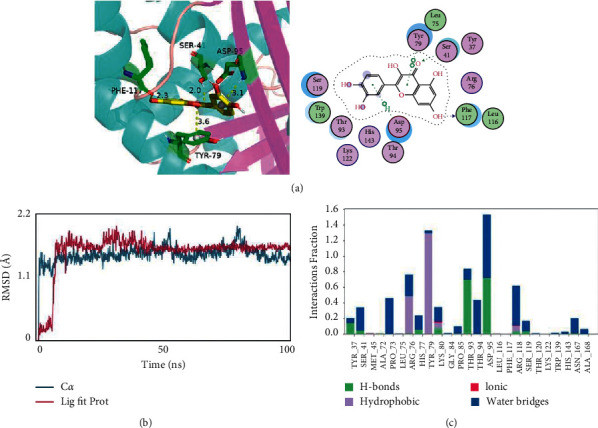
The interactions between quercetin and PAI-1. (a) The binding mode of quercetin with PAI-1. The yellow structure is quercetin, and the green structure represents the binding site of PAI-1. (b) RMSD plot during molecular dynamics simulations of PAI-1 with quercetin. (c) The residues of PAI-1 that interact with quercetin.

Molecular docking between the active compound quercetin and the potential target PAI-1 was performed to explore protein-ligand interactions. The results showed that the binding energy between quercetin and the PAI-1 protein was −7.13 kcal/mol, indicating a strong binding effect (less than −5 kcal/mol). The complex formed by the docked compound and protein was visualized by PyMOL 2.1 software to obtain the binding mode. According to the binding mode (Figure 5(a)), the amino acid residues bound between the compound and protein pocket can be clearly seen. The active pocket of the PAI-1 protein is mainly composed of TYR-79, LEU-75, SER-41, ARGG-76, TYR-37, ASP-95, HIS-143, THR-94, LYS-122, TRP-139, and SER-119 amino acids. Quercetin directly interacts with PAI-1. The amino acid residues involved in the interaction include TYR-79, ASP-95, SER-41, and PHE-117. Quercetin is a pentahydroxyflavone with five hydroxy groups placed at the 3-, 3′-, 4′-, 5-, and 7-positions that can form strong hydrogen bonds and hydrophobic interactions with the pocket amino acids of proteins. For example, quercetin can form hydrogen bonds with SER-41 and PHE-117 amino acids. The hydrogen bond distances are 2.0 Å and 2.3 Å, respectively, which are much smaller than the 3.5 Å distance of the traditional hydrogen bond. Strong binding plays an important role in anchoring small molecules in the protein pocket. In addition, the benzene ring of quercetin also interacts with amino acids TYR-79 and ASP-95 in the active pocket of the protein, forming a strong *π*-*π* conjugate interaction, which plays an important role in stabilizing small molecules. In conclusion, these interactions can improve the stability of quercetin in the PAI-1 protein pocket and form a stable complex with the target protein. Thus, the compound is a potential active small molecule targeting PAI-1.

### 3.8. Molecular Dynamic Analyses

To further study the interaction between the small molecule quercetin and the target protein PAI-1, we used molecular dynamics to simulate the protein-small molecule complex for 100 ns (Figure 5(b)). The stability of RMSD reactive protein and small molecules was evaluated. The greater the RMSD was, the more unstable the complex was. The average RMSD of PAI-1 and the quercetin complex was <2.0 Å, which reaches equilibrium in approximately 10 ns. This result reflects the good combination and stability of the protein and small molecule complex. In addition, the RMSD of small ligand molecules fluctuates slightly near the first 10 ns, which may result from the matching of appropriate conformations after continuous collision with active sites in the protein pocket. Protein-ligand interactions can be monitored throughout the simulation. These interactions can be categorized by type and summarized, as shown in the plot above. Protein-ligand interactions are categorized into four types: hydrogen bonds, hydrophobic, ionic bonds, and water bridges.  Figure 5(c) shows that the small molecule quercetin exhibits good interactions with multiple amino acids in the protein pocket. For example, quercetin has a strong hydrogen bond interaction with THR-93 and ASP-95, and quercetin has an 80% chance of forming a stable hydrogen bond with these two amino acids in the whole molecular dynamics simulation process, indicating that these two hydrogen bonds play an important role in anchoring small molecules in the protein pocket. In addition, quercetin exhibits strong hydrophobic interactions with ARG-76 and TYR-79, especially TYR-79, which plays an important role in stabilizing small molecules.

## 4. Discussion

Currently, the growing number of MDD-induced suicides has become a serious social issue. Although the pathogenesis of MDD is not clearly understood, the last decade has witnessed a step forwards in the diagnosis and treatment of MDD [39]. Accumulating evidence indicates that MDD results from multisystem disorders, including disorders of the nervous system, immune system, and endocrine system [40]. These systems form a complex network through neurotransmitters, endocrine hormones, and cytokines to mediate MDD initiation and development [41]. As a consequence, anti-inflammation and metabolic regulation are promising strategies for MDD treatment. LPS, a bacterial metabolite, is one of the main causes of systemic low-grade inflammation. Exposure to LPS gives rise to a series of mental disorders, such as MDD, cognitive impairment, and social withdrawal [34]. Therefore, LPS is often used to establish animal models of inflammation or MDD. When reaching the blood circulation following intestinal permeability change, LPS causes liver and brain inflammation via a cytokine cascade, which subsequently leads to liver changes, obesity, and metabolic syndrome to mediate MDD [11]. Since the liver is responsible for the clearance of LPS in blood, the enhanced response of the liver to LPS may contribute to the attenuation of inflammation and MDD [42]. Moreover, correlations between LPS and diabetes have been demonstrated. Alison et al. found that type 2 diabetic patients had higher circulating LPS levels (125.4% ↑) than healthy people [43], indicating that diabetes is also a risk factor for inflammation-induced MDD. In fact, patients with diabetes are more susceptible to developing depression than the general population [44]. The pathophysiological mechanisms of diabetes-induced MDD include hyperglycaemia, excess glucocorticoids, inflammation, and insulin resistance [45]. In our present study, the potential antidepressant targets of CR were enriched and identified through GO and KEGG analyses. We found that the regulation of LPS and glucose metabolism may be involved in the antidepressant action of CR. Moreover, AGE-RAGE signalling has been a well-studied cascade involved in various diseases, especially diabetes [46]. Abnormal glucose metabolism increases the activation of NAPDH oxidase and the production of reactive oxygen species (ROS) [47]. AGEs exert deleterious effects in diabetes via interaction with RAGE, thus inducing ROS formation [48]. The combination of AGEs and RAGE elicits oxidative stress, resulting in inflammatory and fibrotic reactions. Therefore, oxidative stress mediated by AGE-RAGE signalling has been recognized as a promising therapeutic target for inflammation-related disorders [49]. Since systemic low-grade inflammation has become the key risk factor for depression, combating AGE-RAGE signalling may represent a potential therapeutic strategy for depression treatment. As a consequence, CR may exert its antidepressant effect by regulating AGE-RAGE signalling in glucose metabolism.

Cyperi Rhizoma, the rhizome of *Cyperus rotundus* L., has been extensively used as medicine and food in Asian countries for centuries [15]. With therapeutic effects on menstrual or emotional disturbances in women, CR has been prescribed in various TCM formulae, such as Xiang-Su-San, Xiang-Fu-Si-Wu decoction, and Chaihu-Shu-Gan-San, to treat neurological disorders [50, 51]. TCM theory states that CR relieves depression and anxiety by dispersing stagnated liver qi to soothe the liver. By studying the mechanism of the anti-MDD effect of CR, we demonstrated that 106 targets corresponding to 15 compounds were involved. The complex connections between CR and MDD indicated that multiple possible mechanisms participate in this process, necessitating further research. This finding also demonstrated that TCM has multiple ingredients, multiple targets, and synergetic effects. The practice of active compound screening from TCM is an important strategy for drug discovery [52]. Natural products exert their pharmacological activity through various novel mechanisms [53]. The exploration of their targets may help us better understand the pathogenesis of multiple diseases. In this research, we attempted to elucidate the active compounds and their molecular targets of CR against MDD. Based on the association between liver function and the metabolism of LPS and glucose, we hypothesized that the anti-MDD effect of CR may be mediated by an altered response of the liver to LPS and glucose metabolism. To verify this hypothesis, we selected DEGs between normal and MDD liver tissue in rats with spontaneous diabetes and found that PAI-1 may represent a potential target of the active compound quercetin in CR. This research provided new insights into MDD treatment from the perspective of the liver. Correlations between MDD and liver disease have been reported. The rate of depression in chronic liver disease is higher than that of the general population [54]. Every third patient with liver cirrhosis or hepatitis shows depressive symptoms [11]. A crucial link between MDD and liver disorder seems to be inflammatory processes. PAI-1 is an inflammatory marker and is mainly synthesized in the liver [28]. Therefore, targeting PAI-1 to combat inflammation may be a useful strategy in the treatment of MDD. Molecular docking and molecular dynamics analyses also confirmed the strong binding of quercetin to PAI-1, indicating that quercetin may represent a promising agent for MDD treatment. However, whether PAI-1 is the precise target of quercetin and the downstream signalling of PAI-1 requires further research.

Some studies demonstrated that elevated PAI-1 levels were found in hippocampal tissues and blood in depressed mice and patients [21, 55], indicating that high PAI-1 levels contribute to the pathogenesis of depression. In contrast, Party et al. found that PAI-1 deficiency induces a depressive-like phenotype, which is associated with reduced serotonin and dopamine levels [56]. This result revealed that the lack of PAI-1 is a factor of predisposition to MDD. According to these facts, we can see that abnormal PAI-1 expression is closely related to depression. The strategy of targeting PAI-1 to combat MDD involves restoring normal PAI-1 expression levels. Excessive inhibition or activation of PAI-1 may both result in adverse outcomes. Given that quercetin is the active compound in Cyperi Rhizoma that combats depression through targeting PAI-1, we predict that quercetin treatment alone may cause a more potent inhibitory effect on PAI-1 than CR treatment. However, safety concerns exist, given that the precise target of quercetin is unknown, and excessive inhibition of PAI-1 may cause side effects. Therefore, quercetin treatment may be more potent but not necessarily more effective than CR treatment. Moreover, there may be other mechanisms for the antidepressant effect corresponding to other active compounds of CR.

In summary, this research may provide evidence for the antidepressant effects of CR through soothing the liver. This study serves as an example for explaining TCM theory through modern pharmacological methods. To emphasize its clinical value and expand its clinical application, further exploration is needed.

## 5. Conclusion

In conclusion, we are the first to report that quercetin is the active compound of CR that acts against MDD by targeting PAI-1 to enhance the liver response to LPS and glucose metabolism, which may shed light on the modern pharmacological mechanism of CR against MDD through soothing the liver. We also demonstrated that PAI-1 is a promising target and that quercetin is a promising agent for MDD treatment, but these findings require further in-depth study.

## Figures and Tables

**Table 1:** Active compounds of CR and their major parameters.

No.	Name	OB	DL
MOL003044	Chryseriol	35.85	0.27
MOL000354	Isorhamnetin	49.6	0.31
MOL003542	8-Isopentenyl-kaempferol	38.04	0.39
MOL000358	*β*-Sitosterol	36.91	0.75
MOL000359	Sitosterol	36.91	0.75
MOL004027	1,4-Epoxy-16-hydroxyheneicos-1,3,12,14,18-pentaene	45.1	0.24
MOL004053	Isodalbergin	35.45	0.2
MOL004058	Khell	33.19	0.19
MOL004059	Khellol glucoside	74.96	0.72
MOL010489	Resivit	30.84	0.27
MOL004068	Rosenonolactone	79.84	0.37
MOL004071	Hyndarin	73.94	0.64
MOL004074	Stigmasterol glucoside_qt	43.83	0.76
MOL004077	Sugeonyl acetate	45.08	0.2
MOL000422	Kaempferol	41.88	0.24
MOL000449	Stigmasterol	43.83	0.76
MOL000006	Luteolin	36.16	0.25
MOL000098	Quercetin	46.43	0.28

**Table 2:** Details of top 5 GO and KEGG enrichment terms.

Ontology	ID	Description	GeneRatio	BgRatio	*P* value	p. adjust	Q value
BP	GO:0032496	Response to lipopolysaccharide	26/106	330/18670	1.12*e* − 22	4.32*e* − 19	1.78*e* − 19
BP	GO:0002237	Response to molecule of bacterial origin	26/106	343/18670	3.01*e* − 22	5.81*e* − 19	2.39*e* − 19
BP	GO:0031667	Response to nutrient levels	28/106	499/18670	2.05*e* − 20	2.64*e* − 17	1.09*e* − 17
BP	GO:0001505	Regulation of neurotransmitter levels	24/106	354/18670	2.03*e* − 19	1.96*e* − 16	8.08*e* − 17
BP	GO:0010038	Response to metal ion	24/106	364/18670	3.88*e* − 19	2.67*e* − 16	1.10*e* − 16
CC	GO:0045121	Membrane raft	20/106	315/19717	3.61*e* − 16	5.23*e* − 14	3.46*e* − 14
CC	GO:0098857	Membrane microdomain	20/106	316/19717	3.84*e* − 16	5.23*e* − 14	3.46*e* − 14
CC	GO:0098589	Membrane region	20/106	328/19717	7.88*e* − 16	7.14*e* − 14	4.73*e* − 14
CC	GO:0099055	Integral component of postsynaptic membrane	12/106	117/19717	1.48*e* − 12	1.00*e* − 10	6.65*e* − 11
CC	GO:0098936	Intrinsic component of postsynaptic membrane	12/106	122/19717	2.45*e* − 12	1.33*e* − 10	8.81*e* − 11
MF	GO:0030594	Neurotransmitter receptor activity	14/106	117/17697	8.72*e* − 15	3.77*e* − 12	2.32*e* − 12
MF	GO:0070405	Ammonium ion binding	11/106	75/17697	7.45*e* − 13	1.61*e* − 10	9.93*e* − 11
MF	GO:0005126	Cytokine receptor binding	15/106	286/17697	1.61*e* − 10	2.32*e* − 08	1.43*e* − 08
MF	GO:0002020	Protease binding	11/106	128/17697	2.85*e* − 10	3.08*e* − 08	1.90*e* − 08
MF	GO:0005125	Cytokine activity	13/106	220/17697	6.83*e* − 10	5.90*e* − 08	3.64*e* − 08
KEGG	hsa04933	AGE-RAGE signalling pathway in diabetic complications	24/101	100/8076	4.48*e* − 25	1.06*e* − 22	3.16*e* − 23
KEGG	hsa05219	Bladder cancer	15/101	41/8076	4.75*e* − 19	5.60*e* − 17	1.67*e* − 17
KEGG	hsa05215	Prostate cancer	16/101	97/8076	3.66*e* − 14	2.88*e* − 12	8.60*e* − 13
KEGG	hsa05418	Fluid shear stress and atherosclerosis	18/101	139/8076	6.42*e* − 14	3.79*e* − 12	1.13*e* − 12
KEGG	hsa05167	Kaposi sarcoma-associated herpesvirus infection	20/101	193/8076	1.70*e* − 13	8.03*e* − 12	2.40*e* − 12

## Data Availability

The data supporting the conclusions of this article are included within the article.
